# Long-Term Anti-Bacterial Immunity against Systemic Infection by *Salmonella enterica* Serovar Typhimurium Elicited by a GMMA-Based Vaccine

**DOI:** 10.3390/vaccines9050495

**Published:** 2021-05-12

**Authors:** Fabio Fiorino, Elena Pettini, Oliver Koeberling, Annalisa Ciabattini, Gianni Pozzi, Laura B. Martin, Donata Medaglini

**Affiliations:** 1 Laboratory of Molecular Microbiology and Biotechnology, Department of Medical Biotechnologies, University of Siena, 53100 Siena, Italy; fiorino4@unisi.it (F.F.); pettini5@unisi.it (E.P.); annalisa.ciabattini@unisi.it (A.C.); gianni.pozzi@unisi.it (G.P.); 2 GSK Vaccines Institute for Global Health S.r.l., 53100 Siena, Italy; OKoeberl@its.jnj.com

**Keywords:** GMMA vaccine, *Salmonella* Typhimurium, salmonellosis, antibody response, cellular immune response, systemic infection

## Abstract

*Salmonella* Typhimurium (STm) represents the most prevalent cause of invasive non-typhoidal Salmonella (iNTS) disease, and currently no licensed vaccine is available. In this work we characterized the long-term anti-bacterial immunity elicited by a STm vaccine based on Generalized Modules of Membrane Antigens (GMMA) delivering O:4,5 antigen, using a murine model of systemic infection. Subcutaneous immunization of mice with STmGMMA/Alhydrogel elicited rapid, high, and persistent antigen-specific serum IgG and IgM responses. The serum was bactericidal in vitro. O:4,5-specific IgG were also detected in fecal samples after immunization and positively correlated with IgG observed in intestinal washes. Long-lived plasma cells and O:4,5-specific memory B cells were detected in spleen and bone marrow. After systemic STm challenge, a significant reduction of bacterial load in blood, spleen, and liver, as well as a reduction of circulating neutrophils and G-CSF glycoprotein was observed in STmGMMA/Alhydrogel immunized mice compared to untreated animals. Taken together, these data support the development of a GMMA-based vaccine for prevention of iNTS disease.

## 1. Introduction

Invasive non-typhoidal Salmonella (iNTS) infections are a serious health concern causing about 535,000 cases per year, of which more than 400,000 were in sub-Saharan Africa, where the highest incidence is observed (34.5 cases per 100,000 person-years) [1]. iNTS is an under recognized leading cause of bacterial bloodstream infections in febrile children and immunocompromised individuals in sub-Saharan Africa, with a high case fatality rate of around 14.5% and up to 39% of community-acquired bloodstream infections [1,2,3,4,5,6]. iNTS infections cause approximately 59,100 global deaths each year, with about 31,200 cases in children under five years [1]. *Salmonella enterica* serovar Typhimurium (STm) and serovar Enteritidis (SEn) are the predominant bacteria associated with iNTS disease in children under five years of age in sub-Saharan Africa [3,4,7,8,9,10].

The higher incidence and increased severity of iNTS disease have been observed also in patients with malaria, anemia, malnutrition, HIV, sickle cell disease, and hemolysis [1,5,10,11,12,13,14]. Although these are critical risk factors for iNTS infection, this under recognized disease is also common in low-HIV-prevalence areas [3,5,13,15].

A key problem with the effective management of invasive Salmonella disease, particularly in Africa, is the lack of appropriate diagnostic tests, the difficulty to distinguish signs and symptoms from other common infections, rapid onset of disease, high mortality, and ineffective antibiotic treatment due to increasing multidrug resistance.

Currently there is no licensed human vaccine against iNTS infections [16,17,18] and the lack of progress relates primarily to the absence of traditional commercial incentives [19,20]. Growing efforts are focused on identifying novel prime-boost strategies and advanced immunization technologies for iNTS vaccine development [11,17,19,21,22,23,24,25,26,27,28,29].

The serovar specific O-antigen portion of STm lipopolysaccharide (O:4,5) has been recognized as an important vaccine target. Bacterial polysaccharides, such as O:4,5, are T-independent type 2 antigens and require conjugation to carrier proteins, such as the non-toxic recombinant form of diphtheria toxin (CRM_197_), tetanus toxoid (TT) or diphtheria toxoid (DT), to induce T-dependent antibody responses [17,25,30,31,32]. Experimental O-antigen-based conjugate vaccines have shown a robust immune response and induced protection against Salmonella challenge in a highly susceptible mouse model [21,23,24,33,34,35].

A promising antigen delivery platform to generate vaccines against Gram-negative bacteria, including Salmonella is the Generalized Modules of Membrane Antigens (GMMA) technology. Gram-negative bacteria naturally shed outer membrane particles into their surrounding environment. This natural phenomenon has been exploited to develop the GMMA-technology. The genetic modifications include the disruption of the links between the outer membrane and the peptidoglycan or inner membrane [e.g., *tolR* knock-out (KO)] [36] to greatly increase release of GMMA. Modification of the lipid A structure of the lipopolysaccharide (LPS), e.g., by deletion of *msbB* and *pagP* genes [37,38], results in decreased innate immune system over-stimulation. GMMA are efficiently, cost effectively and rapidly purified from high density fermentation cultures of the modified bacteria [39,40]. The well characterized GMMA drug substances are then diluted and formulated on Alhydrogel, used as an adsorbant, to reduce residual fever responses in rabbits due to modified lipid A or other PAMPs [39] and generate stable drug products. GMMA do not require further enzymatic or chemical modification, such as the antigens or carrier proteins used for generations of conventional glycoconjugate vaccines. GMMA have an optimal size for immune stimulation, trigger strong innate immune responses and are highly immunogenic at very low doses in animals. *S.* Typhimurium and Enteritidis polysaccharide antigens expressed on GMMA induce serum antibodies with much higher functional activity than corresponding polysaccharide conjugate vaccines or recombinant purified protein antigens [19]. Ease of manufacture and high immunogenicity at low doses make GMMA an innovative technology platform suited for low- and middle-income countries.

A well characterized GMP produced *Shigella sonnei* GMMA candidate vaccine, formulated with Alhydrogel (1790GAHB), was employed in preclinical studies and was highly immunogenic in both mice and rabbits, eliciting a robust antibody response to *S. sonnei* LPS with homologous OAg after single immunization, which were strongly boosted after a second immunization [36,39]. This candidate vaccine, successfully employed for different phase 1 clinical trials in healthy European adults (ClinicalTrials.gov num. NCT02017899, NCT02034500 and NCT03089879), was confirmed to be safe and immunogenic [41,42]. A phase 2a trial using 1790GAHB was conducted in Kenya, a shigellosis-endemic country, enrolling healthy adults (ClinicalTrials.gov num. NCT02676895) [43]. The collective safety and immunogenicity results support further evaluation of GMMA-based vaccines for other diseases and in younger individuals from developing countries.

The aims of this study were to: (i) characterize the long-term kinetics of STm O:4,5 antibody response elicited by STmGMMA/Alhydrogel; (ii) study the cellular immune response after immunization; and (iii) evaluate the impact of the candidate vaccine against systemic infection by STm.

## 2. Materials and Methods

### 2.1. STmGMMA/Alhydrogel

The STmGMMA, their formulation on Alhydrogel and material for this study were provided by GSK Vaccines Institute for Global Health (Siena, Italy). Briefly, STmGMMA were prepared from genetically modified *S.* Typhimurium strain NVGH2363 (Δ*tolR*, Δ*msbB* and Δ*pagP*). NVGH2363 was routinely fermented in batch mode at 25 L scale and STmGMMA (2363-GMMA), released into the fermentation broth, were purified using two consecutive tangential flow filtration (TFF) steps: a microfiltration in which the culture supernatant containing the GMMA is separated from the bacteria, and an ultrafiltration, in which the GMMA were separated from soluble proteins and nucleic acids. A final concentration of the purified GMMA was performed to obtain the required concentration for the formulation process and sterile filtered. STmGMMA, without further modification, were adsorbed to aluminum hydroxide (Alhydrogel, Brenntag, Frederikssund, Denmark) to produce STmGMMA/Alhydrogel. Formulations were prepared containing 100 µg/mL STmGMMA in OAg and 0.35 mg Al^3+^/mL, without the addition of preservative.

### 2.2. Animals

Six-weeks old female C57BL/6 mice were purchased from Charles River (Lecco, Italy) and treated according to national guidelines (Decreto Legislativo 26/2014). Animals were housed under specific pathogen-free conditions in the animal facility of the Laboratory of Molecular Microbiology and Biotechnology (LA.M.M.B.), Department of Medical Biotechnologies at University of Siena, in cages provided with food and water ad libitum. Experiments were planned and conducted utilizing the three R’s principles (reduce, replace, and refine), which included environmental enrichment and nesting, veterinary oversight, numbers reflecting statistical significance and the use of anesthesia followed by cervical dislocation for the sacrifice. All animal studies were approved by the Italian Ministry of Health with authorization n° 691/2017-PR and n° 371/2019-PR.

### 2.3. Immunizations

**Figure 1:**
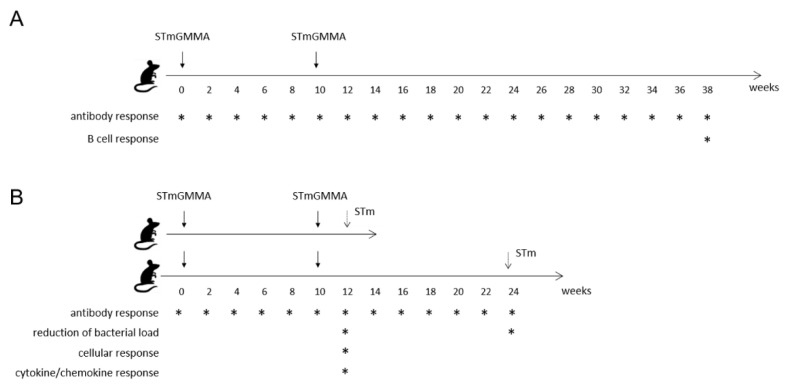
Experimental design for immunogenicity and challenge studies in mice. C57BL/6 mice were immunized at 0 and 10 weeks (solid vertical arrows) with STmGMMA/Alhydrogel, performed by subcutaneous (SC) route, using the dose of 10 µg STmGMMA quantified based on O-antigen. (**A**) The antibody responses were monitored in sera and fecal samples every two weeks for 38 weeks, and B cell response in spleen and bone marrow was studied at the sacrifice of mice at week 38. (**B**) Mice were challenged intravenously (IV) with 10^4^ CFU of the virulent STm D23580 at week 12 or 24 (dashed vertical arrows) and the bacterial load was evaluated 24 h after challenge. Cellular response and cytokine/chemokine production were analyzed 24 h after challenge performed at week 12. * indicates the analysis performed for each time point.

Groups of C57BL/6 mice were immunized at weeks 0 and 10 with STmGMMA/Alhydrogel (Figure 1A). Immunizations were performed by subcutaneous (SC) route at the base of the tail using the dose of 10 µg STmGMMA quantified based on O:4,5 antigen, in a volume of 100 µL. Control groups were injected with saline in a volume of 100 µL.

### 2.4. Challenge Model

*S.* Typhimurium ST313 strain D23580, clinical isolate from Malawi [44,45], was used to infect mice. Bacteria were grown in Luria-Bertani (LB) medium [Tryptone (10 g/L, Oxoid, Basingstoke, UK); Sodium Chloride (10 g/L, Carlo Erba, Milan, Italy); Yeast extract (5 g/L, Oxoid)] up to optical density (OD) 590 = 0.25, and stored at −80 °C with 10% glycerol (Carlo Erba). Solid medium was prepared by addition of 1.5% agar (Oxoid) to LB broth. Counts of colony forming units (CFU) were performed on LB agar at 37 °C with 5% CO_2_.

Groups of C57BL/6 mice were challenged by intravenous (i.v.) injection of 10^4^ CFU of STm D23580 in a volume of 60 µL of LB medium, at weeks 12 or 24, and sacrificed 24 h later (Figure 1B). Sacrifice was performed by lethal anesthesia with intraperitoneal injection of Tiletamine Hydrochloride/Zolazepam (Zoletil 50/50, 30 mg/kg, Laboratoires Vibrac, Carros, France) and Xilazina (Rompun, 8 mg/kg, Bayer Spa, Leverkusen, Germany) and cervical dislocation. The challenge dose was selected based on dose-response infection studies using three different doses of bacteria for the i.v. infection (10^3^, 10^4^ and 10^5^ CFU/mouse), and evaluating the weight loss of animals, the clinical score based on a scale for severity of disease, from 0 (normal) to 5 (moribund), and the bacterial load in different compartments.

### 2.5. Samples Collection and Treatment

The long-term immune response was studied in groups of C57BL/6 mice sacrificed at week 28 after the second immunization with STmGMMA/Alhydrogel. One day after challenge, sera, fecal samples and intestinal washes were collected from each mouse to study the kinetics and persistence of antibodies; blood, spleen and bone marrow were collected to study the cellular immune response, and blood, spleen and liver were used to study the bacterial clearance.

For the study of humoral response, blood and fecal samples were collected on weeks 0, 2, 4, 6, 8, 10, 12, 14, 16, 18, 20, 22, 24, 26, 28, 30, 32, 34, 36, and 38, while intestinal washes were collected at the sacrifice, at week 12. Blood was taken from the temporal plexus, incubated for 30 min at 37 °C and centrifuged at 1200× *g* at 4 °C for 15 min and the sera collected. Fecal samples from individual mice were weighed and gently dissolved at 100 mg/mL in PBS–1% BSA (both from Sigma-Aldrich, St. Louis, MO, USA), centrifuged at 15,000× *g* at 4 °C for 10 min, and 1% (*v*/*v*) protease inhibitor cocktail (Sigma-Aldrich) was added to supernatants. To perform intestinal washes, the small intestine was removed and washed with 1 mL of PBS-1% BSA three times. Samples were centrifuged at 10,000× *g* at 4 °C for 10 min and 1% (*v*/*v*) protease inhibitor cocktail (Sigma-Aldrich) was added to supernatants. Erythrocyte contamination was estimated in intestinal samples using the Hemoplus (Sarstedt, Nümbrecht, Germany) kit and results were too low to account for the observed intestinal antibody response. All sera, fecal and intestinal samples were stored at −80 °C until ELISA analysis.

For the study of cellular immune response, samples were collected at the sacrifice of animals. Blood was collected by cardiac puncture in a tube with heparin (5000 U/mL, Athena Pharma Italia, Rome, Italy), then added to equal amount (200 µL/sample) of Hanks’ Balanced Salt Solution (HBSS; Sigma-Aldrich) and finally stratified over Hystopaque 1119 (Sigma-Aldrich) according with the manufacturer’s protocol. Samples were centrifuged at 700× *g* at room temperature for 30 min, without applying brake. After washes in PBS, red blood cells (RBC) were removed from samples using RBC lysis buffer, according with the manufacturer’s protocol (eBioscience, San Diego, CA, USA). Spleen and bone marrow samples were collected in tubes with complete medium [cRPMI, RPMI 1640 supplemented with 10% (*v*/*v*) fetal bovine serum (both from Gibco, Grand Island, NY, USA), 100 U/mL penicillin and 100 µg/mL streptomycin (Sigma-Aldrich)]. Cell from bone marrow were collected by removing the muscles and residue tissues surrounding the femur, eliminating epiphyses, and flushing out the bone marrow with cRPMI by using a syringe with a 27 G needle. All organs were mashed through 70 µm nylon screens (Sefar Italia, Torino, Italy), washed two times in cRPMI, and erythrocytes were removed. The number of cells was defined using the automated cell counter (Bio-Rad Laboratories, Hercules, CA, USA).

For evaluation of bacterial load, blood, spleen and liver samples were aseptically collected in tubes with LB with 10% glycerol, homogenized using Stomacher 80 (Seward Ltd., Worthing, UK) and stored at −80 °C.

### 2.6. Enzyme-Linked Immunosorbent Assay (ELISA)

Serum antibodies: Well characterized STm O:4,5 polysaccharide (NVGH2850) [19] was provided by GVGH and used as plate coating antigen for detection of specific serum IgM, IgG and IgA. Maxisorp microtiter plates (Nunc, Roskilde, Denmark) were coated with O:4,5 (5 µg/mL; 100 µL/well) in a carbonate buffer pH 9.4 overnight at 4 °C. Coating was removed and plates were blocked with 200 µL/well of PBS—0.05% Tween 20 (Sigma-Aldrich)–5% fat-free milk (AppliChem, Darmstadt, Germany) for 1 h at room temperature. Plates were washed with PBS–0.05% Tween 20 and serum samples were added and titrated in twofold dilution in duplicate in PBS—0.05% Tween 20—0.1% BSA (diluent buffer) in 100 µL/well. After incubation for 2 h at room temperature, plates were washed, incubated for 1 h at room temperature with the alkaline phosphatase-conjugate goat anti-mouse IgM, IgG or IgA (all diluted 1:1000 and from Southern Biotechnology, Birmingham, AL, USA) in 100 µL/well and developed by adding 1 mg/mL of alkaline phosphatase substrate (Sigma-Aldrich) in 100 µL/well. The optical density was recorded using Multiskan FC Microplate Photometer (Thermo Scientific, Waltham, MA, USA). Antibody end point titres were expressed as the reciprocal of the sample dilution reporting double the background OD value.

Mucosal antibodies: Anti-O:4,5 IgG and IgA in fecal samples and intestinal washes were determined by ELISA, as previously described [46]. As the concentration of mucosal IgG and IgA is variable, the amount of anti-O:4,5 IgG and IgA was normalized to the total IgG and IgA concentration in each sample. Total IgG and IgA were determined on flat bottom maxisorp microtiter plates coated with anti-mouse IgG and IgA (1 µg/mL, Southern Biotechnology), while anti-O:4,5 IgG and IgA were assayed as described above, using plate coating antigen provided by GVGH. Samples were tested in duplicate two-fold dilutions and plates incubated overnight at 4 °C. The concentration of total and anti-O:4,5 IgG and IgA were calculated against a dilution curve of mouse myeloma standard IgG and IgA (Southern Biotechnology) included on the same plate. Results were expressed as µg of anti-O:4,5 IgG or IgA per mg of total IgG or IgA.

### 2.7. Serum Bactericidal Activity (SBA) Assay

Individual mouse sera collected from groups sacrificed at week 12 were tested for SBA assay, as previously described [47]. *S.* Typhimurium D23580 strain was used as target strain against STm, to perform SBA reactions [34]. Bacteria was grown in Luria Bertani (LB) medium to log-phase (OD: 0.2), diluted 1:30,000 in PBS to approximately 3 × 10^3^ colony forming units (CFU)/mL and distributed into sterile polystyrene U bottom 96-well microtiter plates (12.5 µL/well). Sera samples serially diluted 2 or 3-folds (starting from 1:100 dilution), were added to each well (final volume 50 µL, ~620 CFU/mL). Sera were heat-inactivated and Baby Rabbit Complement (Cederlane, Burlington, Canada) was used at 50% of the final volume. Each sample was tested in triplicate on three different days. One hundred microliters reaction mixtures from each well were spotted on LB-agar plates at time zero (T0) to assess initial CFU, and at 3 h (T180) after incubation at 37 °C. LB-agar plates were incubated overnight at 37 °C and the resulting CFU were counted the following day. Bactericidal activity was determined as serum dilutions necessary to obtain 50% CFU reduction at T180 compared with T0. Serum titres equal to 1 were designated when no bactericidal activity was detected.

### 2.8. Multiparametric Flow Cytometric Analysis

Cells from spleen, bone marrow and blood were used to characterize B and T cell responses induced by immunization and presence of different cell populations. Samples were incubated for 30 min at 4 °C in Fc-blocking solution [cRPMI with 5 μg/mL of CD16/CD32 mAb (clone 93; eBioscience)]. Cells were then washed and surface stained with different combination of antibodies: BUV395-conjugated anti-CD3 (clone 145-2C11), BUV737-conjugated anti-CD19 (clone1D3) and BV510-conjugated anti-IgD (11-26c.2a), AF700-conjugated anti-CD45R (B220, clone RA3-6B2), FITC-conjugated anti-GL7 (clone GL-7), PE-Cy7-conjugated CD95 (clone Jo2), BV421-conjugated anti-TACI (clone 8F10), PE-conjugated anti CD138 (clone 281-2), BB700-conjugated anti-CD38 (clone 90/CD38), BV650-conjugated anti-IgM (clone II/41), BV605-conjugated anti-IgG1 (clone A85-1), PE-CF594-conjugated anti-CXCR4 (clone 2B11/CXCR4), AF647-conjugated anti-CD73 (cloneTY/239), BV786-conjugated anti-CD45 (clone 30-F11), BV510-conjugated anti-CD3 (clone 145-2C11), FITC-conjugated anti-Ter119 (clone Ter119), APC-conjugated anti-CD11b (clone M1/70), APCR700-conjugated anti-CD11c (clone N418), PE-conjugated anti-MHC-II (clone M5/114.15.2), Pe-Cy7-conjugated anti-Ly6G (clone 1A8), BV421-conjugated anti-Ly6C (clone AL-21), PE-CF594-conjugated anti-F4/80 (clone T45-2342), BV650-conjugated anti-B220 (clone RA36B2), BV605-conjugated anti-NKp46 (clone 29A1,4), all from BD Biosciences (New York, NY, USA). At the end of the staining, cells were washed twice in PBS and then labeled with live/dead Fixable Viability Stain 780 according to the manufacturer instruction (BD Biosciences). Cells from different samples were washed twice in PBS, Fix Perm was then added for 20 min at 4 °C, and cells were resuspended in 250 µL in filtered PBS. All antibodies were titrated before use to determine the optimal dilution. Fluorescence-minus-one controls were used to detect the background fluorescence for each fluorochrome. About 7 × 105 events were acquired and stored for each sample with SO LSRFortessa X20 flow cytometer (BD Biosciences). Data analysis was performed using FlowJo v10 (TreeStar, Ashland, OR, USA).

### 2.9. Memory B Cell ELISpot Assay

Spleen cells collected 38 weeks after primary immunization were evaluated for IgM and IgG production using Murine IgM/IgG Double-Color Enzymatic ELISpot assay (CTL Europe GmbH, Bonn, Germany). The protocol was performed according to the manufacturer instruction.

Cells (4 × 10^6^/mL) were pre-stimulated with a polyclonal stimulus (B-Poly-S, diluted 1:1000) in CTL-Test B medium for 4 days, at 37 °C with 5% CO_2_. Multiscreen filter (PVDF) 96 well plates (CTL Europe GmbH) were coated with 80 µL/well of O:4,5 (10 µg/mL) or Murine Ig Capture Ab. After overnight incubation at 4 °C, coated wells were washed with PBS, and 50 µL of CTL-Test B medium supplemented with 1% L-glutamine (Sigma-Aldrich) were added to each well. 1 × 10^6^ pre-stimulated cells were seeded in a volume of 50 µL/well of CTL-Test B medium and incubated overnight at 37 °C in the presence of 5% CO_2_. Cells were then removed by washing with PBS-0.05% Tween 20, and anti-murine IgM/IgG Detection Solutions were added for 3 h at room temperature. Plates were washed, incubated with 80 µL/well of Tertiary Solution (FITC-HRP and Strep-AP, both diluted 1:1000) at room temperature for 1 h, washed again and Blue and Red Developer Solutions were added, each for 15 min. The reaction was stopped by extensive washing in tap water, and plates were dried in the dark at room temperature. The number of spots was determined by plate scanning and analysis services performed with an Immunospot S6 Ultimate Analyzer (CTL Europe GmbH).

### 2.10. Multiplex Cytokine and Chemokine Assay

IL-1α, IL-1β, IL-2, IL-3, IL-4, IL-5, IL-6, IL-9, IL-10, IL12p40, IL-12p70, IL-13, IL17A, eotaxin, G-CSF, GM-CSF, IFN-γ, KC, MCP-1, MIP-1α, MIP-1β, RANTES and TNF-α production was assessed by Luminex immunoassay in sera samples. Sera samples were collected and stored at −80 °C. Analytes were detected using the BioPlex pro mouse cytokine group 1—panel 23-plex immunoassay (Bio-Rad) following the manufacturer’s protocol and analyzed by Bio-Plex Magpix Multiplex Reader (Bio-Rad). Cytokine and chemokine concentrations were expressed as pg/mL and were calculated based on standard curve data using Bio-Plex Manager 6.1.

### 2.11. Statistical Analysis

All samples were tested individually. The amount of serum antibodies was expressed as geometric mean titers (GMT) ± 95% CI, while the amount of antibodies in intestinal samples were reported as concentration (µg of specific per mg of total IgG) ± SEM. Statistical analysis of antibody titers was performed on log-transformed data. An unpaired Mann–Whitney test was used for analyzing antibody amounts at two different time points and for statistical differences between two independent groups. The correlation between the antibodies in sera and fecal samples, serum GMT and ASCs or CFUs, in each animal was performed calculating Pearson’s correlation coefficient, r. Statistical significance was defined as *p* ≤ 0.05. Statistical analyses were performed using Graph Pad Prism v. 9 (GraphPad Software, San Diego, CA, USA).

## 3. Results

In the present work, for the first time, the long-term immune response to STmGMMA/Alhydrogel and its ability to reduce the bacterial load in different compartments after systemic challenge was extensively characterized. C57BL/6 mice were immunized twice subcutaneously, 10 weeks apart, with 10 µg STmGMMA/Alhydrogel quantified based on O-antigen. O:4,5-specific IgM and IgG antibody responses in serum as well as their in vitro bactericidal activity, and IgG in intestinal samples were assessed. O:4,5-specific memory B cells and long-lived B-cells were also evaluated in spleen and bone marrow after STmGMMA/Alhydrogel immunization. After systemic challenge with a virulent STm strain, the reduction of the bacterial load in different compartments, the decrease of neutrophils in blood and the modulation of different cytokine/chemokine production were further analyzed in mice immunized with STmGMMA/Alhydrogel compared to untreated animals.

### 3.1. Serum Antibody Response

O:4,5-specific IgM, IgG and IgA antibody responses following STmGMMA/Alhydrogel immunization were evaluated by ELISA in serum samples collected every two weeks for 38 weeks (Figure 1A).

**Figure 2:**
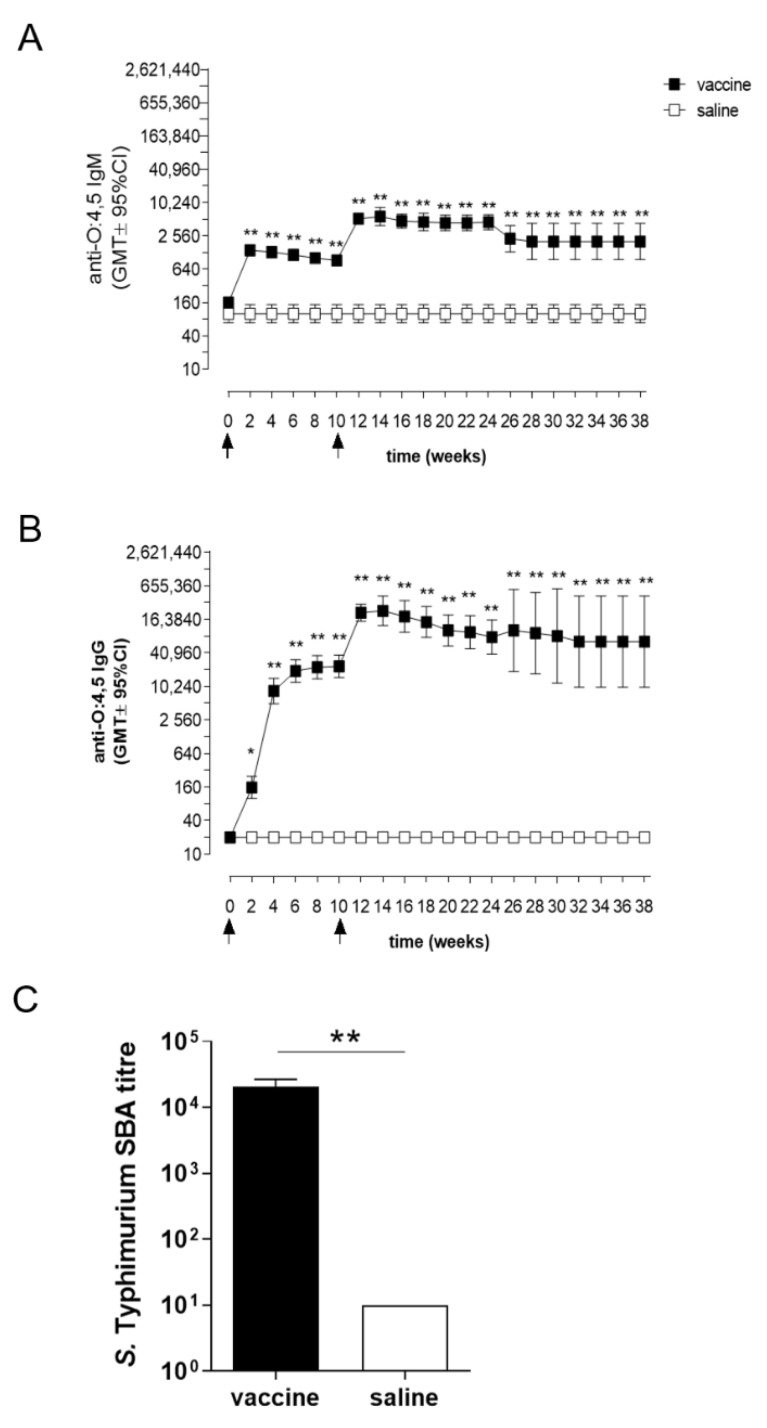
Time course of *S.* Typhimurium O:4,5 specific serum IgM and IgG response and SBA. C57BL/6 mice were immunized at 0 and 10 weeks (arrows) with STmGMMA/Alhydrogel, administered by the SC route. Control group of mice receiving saline administration was included. O:4,5-specific IgM (**A**) and IgG (**B**) titers were evaluated by ELISA before immunization (day 0) and every 2 weeks for 38 weeks. Data are reported as geometric mean titers (GMT) ± 95% CI from 4 different experiments with a total of 36 mice. (**C**) SBA on single sera collected at week 12 and tested against *S*. Typhimurium D23580 isolates. Bactericidal activity was determined as the dilution necessary to obtain 50% CFU reduction at T180 compared with T0. Bars indicate the mean ± SEM of 6 mice per group. Unpaired Mann-Whitney test was used to assess statistical difference between vaccinated and control groups. * *p* ≤ 0.05 and ** *p* ≤ 0.01 refer to immunized versus saline.

Two weeks after priming, STmGMMA/Alhydrogel induced a significantly higher O:4,5-specific IgM response compared to control group at all sampling points (*p* ≤ 0.01; Figure 2A); IgM levels were maintained until the booster immunization at week 10. Boosting induced a significant increase of O:4,5-specific IgM (*p* ≤ 0.01 week 10 vs. 12; Figure 2A), which reached the highest GMT of 5750 at week 14 (*p* ≤ 0.01; Figure 2A); significant anti-O:4,5 IgM levels were maintained for about 9 months (week 38).

A significant progressive increase was observed also for O:4,5-specific serum IgG, that maintained a mean titer of about 20,480 for 10 weeks after priming (*p* ≤ 0.01 vs. control; Figure 2B). The booster immunization further increased the level of O:4,5-specific IgG (*p* ≤ 0.05 week 10 vs. 12), and anti-O:4,5 IgG persisted with a range of GMT between 65,020 and 231,705 until the end of the study (*p* ≤ 0.01; Figure 2B).

Two weeks after boosting (week 12), the functional activity of serum antibodies against *S.* Typhimurium D23580 was assessed in mice immunized with STmGMMA/Alhydrogel. A statistically significant higher SBA titer (*p* < 0.001) was observed in the vaccine group compared to either saline or Alhydrogel injected mice (Figure 2C).

No statistically significant difference in O:4,5-specific serum IgA was observed in sera collected at different time points between STmGMMA/Alhydrogel and saline group (data not shown).

Taken together, these data show the ability of STmGMMA/Alhydrogel immunization to elicit rapid and high level O:4,5-specific serum IgM and IgG antibodies capable of persisting up to 10 weeks after a single immunization and seven months after boosting (week 38). This is the first report of long-term kinetics of humoral response elicited by STmGMMA/Alhydrogel, and further supports the GMMA-technology as an appropriate vaccine platform for use in resource poor settings.

### 3.2. Intestinal Antibody Response

The intestinal anti-O:4,5 IgG and IgA responses were evaluated by ELISA in fecal samples collected every two weeks for the 38 weeks of the study. For each sample, the amount of O:4,5-specific IgG was normalized to the total IgG concentration, in order to minimize potential sample variations.

**Figure 3:**
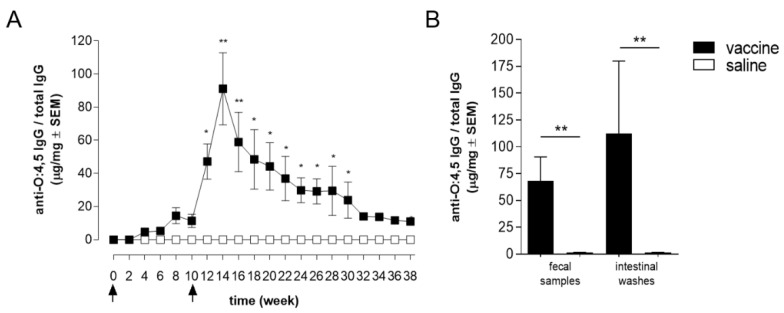
Time course of S. Typhimurium O-antigen-specific IgG in the intestine. C57BL/6 mice were immunized at 0 and 10 weeks (arrows) with STmGMMA/Alhydrogel, administered by the SC route. Control group of mice receiving saline administration was included. (**A**) O:4,5-specific IgG in fecal samples evaluated by ELISA before immunization (day 0) and every 2 weeks for 38 weeks. Data are reported as mean concentration (µg of specific IgG/mg of total IgG) ± SEM from samples collected in 4 different experiments with a total of 36 mice. (**B**) O:4,5-specific IgG in fecal samples and in intestinal washes evaluated at week 12 in samples from two different experiments, each with 6 mice per group. Unpaired Mann-Whitney test was used to assess statistical difference between vaccinated and control group. * *p* ≤ 0.05 and ** *p* ≤ 0.01 refer to immunized versus saline.

After STmGMMA/Alhydrogel primary immunization, a slight increase of O:4,5-specific IgG was observed in fecal samples of immunized mice compared to controls (Figure 3A). The booster immunization induced a significant increase of anti-O:4,5 IgG response, that peaked 4 weeks later (week 14, *p* < 0.01 between STmGMMA/Alhydrogel and saline, Figure 3A). The level of O:4,5-specific IgG slowly decreased until the sacrifice at week 38, with a statistically significant difference between immunized and control group maintained from week 12 until week 30 (*p* < 0.05, Figure 3A).

Interestingly at each time point, a positive correlation between serum and fecal O:4,5-specific IgG was observed following vaccination with STmGMMA/Alhydrogel [Pearson’s correlation r = 0.97 (*p* ≤ 0.01) at week 12 and r ≥ 0.90 (*p* ≤ 0.05) for time points from week 14 to 30].

The presence of IgG was also studied in intestinal washes collected at sacrifice, two weeks after boosting (Figure 3B), and a positive correlation was observed between intestinal washes and fecal samples (r = 0.97). These data underline the critical importance of fecal samples as a tool to monitor in vivo intestinal immune response, avoiding the sacrifice of animals.

No statistically significant difference in O:4,5-specific IgA was observed in fecal samples collected at different time points between STmGMMA/Alhydrogel and saline group (data not shown).

### 3.3. B Cell Response

**Figure 4:**
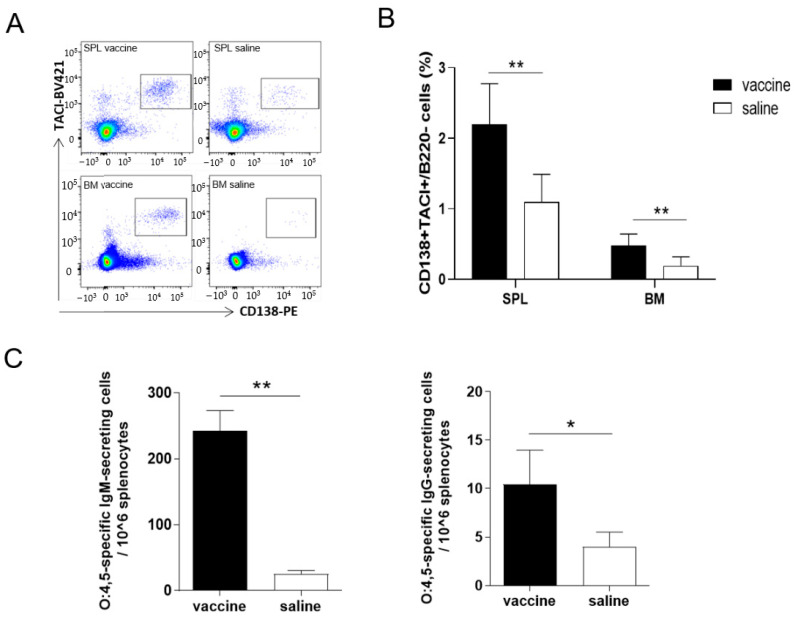
B cell response. C57BL/6 mice were immunized at 0 and 10 weeks with STmGMMA/Alhydrogel, administered by the SC route, and sacrificed at week 38. Control group of mice receiving saline administration was included. (**A**) Representative dot plot of CD138 and TACI staining PCs detected in spleen and bone marrow of STmGMMA/Alhydrogel immunized mice compared to control mice. Cells are gated on B220-IgD-CD3-live cells. (**B**) Frequencies of positively stained CD138+TACI+ PCs with respect to B220- in spleen (SPL) and bone marrow (BM) of STmGMMA/Alhydrogel immunized mice compared to control mice assessed with flow cytometric analysis. Bars indicate the mean ± SEM of 6 mice for each group. (**C**) Amount of O:4.5-specific IgM and IgG-ASCs per million splenocytes. Bars indicate the mean ± SEM of 6 mice for each group. Statistical analysis was performed by using unpaired Mann-Whitney test. * *p* ≤ 0.05 and ** *p* ≤ 0.01.

As B cells are key players in the vaccine induced immune response, the quality and quantity of the B-cell response was examined in spleen and bone marrow samples, by multiparametric flow cytometry and ELISpot assay. Twenty-eight weeks after the booster immunization with STmGMMA/Alhydrogel, the presence of long-lived plasma cells (PCs) was assessed in the spleen and bone marrow by measuring the frequency of IgD^-^B220^-^CD138^+^TACI^+^ cells (Figure 4A). PCs were significantly higher in both spleen and bone marrow samples of mice immunized with the STmGMMA/Alhydrogel (2.2% and 0.5% respectively), compared to mice receiving saline (Figure 4B; *p* < 0.01).

The induction of antigen-specific memory B cells was demonstrated using a dual-color ELISpot assay, in which cells secreting both IgM and IgG (ASCs) specific for O:4,5 antigen were detected among reactivated memory B cells. Significant levels of both O:4,5-specific IgM and IgG secreting cells were observed in the spleen of immunized mice compared to control group (IgM with 242 spots/10^6^ splenocytes vs. 24 spots, *p* < 0.01; IgG with 11 spots/10^6^ splenocytes vs. 4 spots, *p* < 0.01; Figure 4C). Taken together, these results show that STmGMMA/Alhydrogel induced a robust and long-lasting B-cell response, characterized by long-lived plasma cells, memory B cells, and circulating antibodies. This is the first report of comprehensive characterization of memory B cell generation as a result of GMMA vaccination. These data corroborate the observed persistent serum antibody responses reported above.

### 3.4. Impact of STmGMMA/Alhydrogel Immunization on Challenge Outcomes

**Figure 5:**
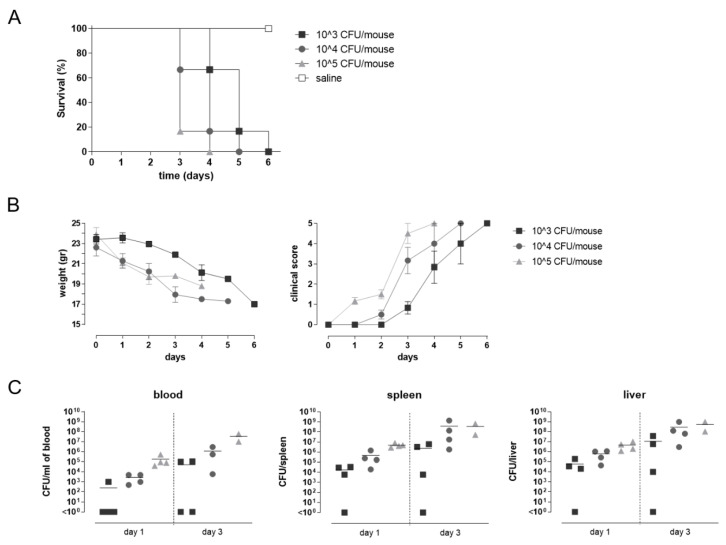
Murine systemic challenge model. C57BL/6 mice were infected by the IV route with 10^3^, 10^4^ or 10^5^ CFU/mouse of the virulent strain STm D23580. As control, a group received saline. (**A**) Kaplan-Meier curve of mouse survival. Results were expressed as percentage of survival over time using 6 mice for each group. (**B**) Evaluation of the animal body weight expressed in grams, and the clinical score analysis based on a scale for severity of disease, from 0 (normal) to 5 (moribund). Data are reported as mean value ± SEM for each time point using 6 mice for each group. (**C**) Bacterial load in blood, spleen and liver samples collected 1 or 3 days after infection. Values are reported as the number of CFU/mL of blood or CFU/organ of surviving mice for each group. Symbols represent individual mice and bars indicate the mean CFU for each infection dose.

The impact of STmGMMA/Alhydrogel immunization was evaluated following systemic infection of mice with 10^4^ CFU/mouse with the invasive STm D23580 strain (Figure 1B). The D23850 strain is a member of the ST313 sequence type clade, which is the primary cause of iNTS disease across Africa [2]. Murine intravenous challenge was used to model a systemic infection due to the capacity of STm D23580 to cause bacteremia infection in humans, that is the hallmark of febrile illness in young children presenting with iNTS disease. The murine model of intravenous infection using the STm D23580 strain was set up in C57BL/6 mice, using three different doses of bacteria (10^3^, 10^4^ and 10^5^ CFU/mouse). For each mouse, survival (Figure 5A), weight loss and clinical score based on a scale for severity of disease of infected animals (Figure 5B), and the bacterial load reduction in blood, spleen, and liver at both 24 and 72 h post infection (Figure 5C) were collected and the group responses analyzed.

The dose of 10^4^ CFU/mouse was selected for further studies in mice immunized with STmGMMA/Alhydrogel due to the gradual disease progression and bacterial dissemination in different compartments. At this bacterial dose, the time point of 24 h after infection was identified as the most appropriate to evaluate the impact of immunization on the reduction of both bacterial load and weight loss, and to analyze the modulation of the early innate response in blood, ensuring the survival of 100% of infected mice according to 3R’s principles.

**Figure 6:**
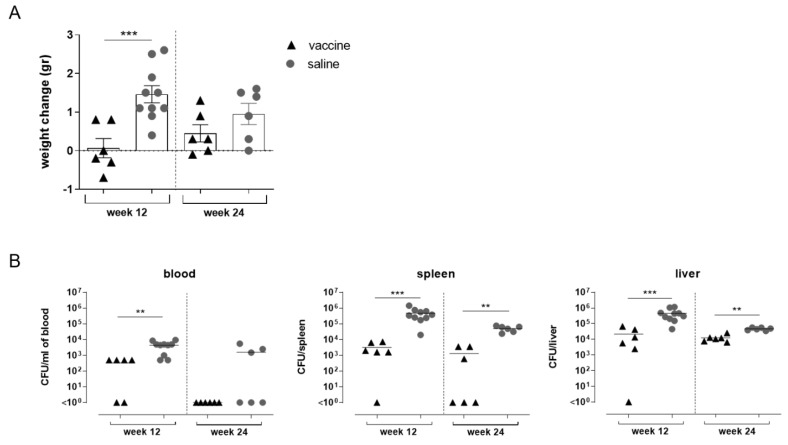
In vivo impact of STmGMMA/Alhydrogel immunization on STm challenge outcomes. Groups of C57BL/6 mice were immunized with STmGMMA/Alhydrogel by the SC route at weeks 0 and 10, and IV challenged with STm at week 12 or 24. A control group received saline administration. Mice were sacrificed 1 day after infection. (**A**) Weight loss (in grams) detected 24 h after infection of STmGMMA/Alhydrogel immunized or saline injected. Symbols represent individual mice and bars indicate the mean ± SEM of 6 or 10 mice per group. (**B**) Bacterial load detected in blood, spleen and liver samples collected at sacrifice. Values are reported as the number of CFU/mL of blood or CFU/organ of 6 or 10 mice per group; symbols represent individual mice and bars indicate the mean CFU for each treatment. Unpaired Mann-Whitney test was used to assess statistical difference between groups for each time point. ** *p* ≤ 0.01 and *** *p* ≤ 0.001.

As shown in Figure 6A, mice immunized twice with STmGMMA/Alhydrogel and challenged with STm D23580 at week 12 (2 weeks after boost) showed a significantly smaller change in weight compared to saline group (*p* = 0.0009; Figure 6A).

Immunized mice also showed a significantly lower bacterial load in blood, spleen and liver samples 24 h after infection compared to saline injected animals (week 12 with *p* ≤ 0.01; Figure 6B). A similar significant lower bacterial load was also observed in spleen and liver when mice were challenged at week 24 (14 weeks after booster immunization with *p* ≤ 0.01; Figure 6B). The reduced bacterial load in these compartments represents an important biomarker to evaluate the impact of STmGMMA/Alhydrogel immunization on experimental challenge outcomes. A positive correlation was observed between higher O:4,5 specific IgG in serum and the bacterial CFU in the spleens of STmGMMA/Alhydrogel immunized and STm challenged mice (r = 0.947; *p* = 0.04) (data not shown).

### 3.5. Characterization of Blood Cell Populations

**Figure 7:**
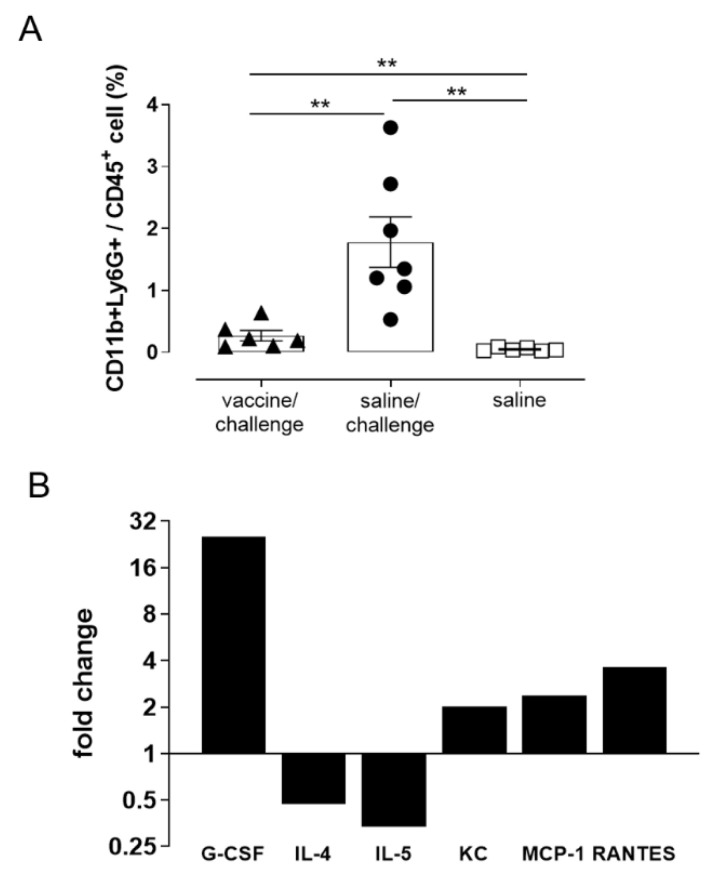
Neutrophil count and cytokines/chemokines in blood of STm challenged mice. C57BL/6 mice were immunized with STmGMMA/Alhydrogel by the SC route at weeks 0 and 10, and IV challenged with STm at week 12. Mice were sacrificed 24 h after infection. As control, two groups received saline administration and the first was challenged with STm, while the other was left unchallenged. (**A**) Neutrophil population in blood identified as CD45+CD11b+Ly6G+ cells by multiparametric flow cytometry analysis. Symbols represent individual mice and bars indicate the mean ± SEM of 6 or 7 mice per group. Unpaired Mann-Whitney test was used to assess statistical difference between groups. ** *p* ≤ 0.01. (**B**) Fold change of cytokine/chemokine concentration detected in mice challenged with STm compared with mice immunized with STmGMMA/Alhydrogel and then infected. Data are reported as arithmetic mean of 6 animals for each group.

The innate immune cell response in blood of STmGMMA/Alhydrogel immunized mice was characterized 24 h after STm challenge (Figure 1B). The frequency of monocytes (CD11b+Ly6C+), macrophages (CD11b+F4/80+), dendritic cells (CD11c+MHCII+) and NK cells (Nkp4.6+) was similar between saline injected and immunized mice (data not shown) however, the frequency of CD11b+Ly6G+ neutrophils was significantly lower in immunized mice compared to saline injected mice shortly after bacterial challenge (1.8% and 0.3% respectively; *p* < 0.01; Figure 7A). The frequency of neutrophils detected among immunized and challenged mice was similar to that observed in mice immunized but not challenged (data not shown).

The data reported indicate that STmGMMA/Alhydrogel vaccination controls Salmonella infection and the associated inflammatory phenomena including neutrophils recruitment.

### 3.6. Analysis of Cytokine and Chemokine Production

The modulation of serum cytokines and chemokines was studied by Luminex immunoassay at week 12 in immunized and challenged mice (Figure 1B). Figure 7B reports the fold change of six cytokines/chemokines, among 23 different secreted molecules analyzed, that were modulated with at least a two-fold change between saline treated and STmGMMA/Alhydrogel immunized mice subsequently challenged with STm. Following STm challenge, serum samples from saline treated mice showed higher concentrations of G-CSF, KC, MCP-1 and RANTES and lower amounts of IL-4 and IL-5 compared to STmGMMA/Alhydrogel immunized mice. A positive correlation was observed between increased levels of G-CSF and KC, two of the major chemoattractants responsible for recruiting neutrophils, and the increased frequency of circulating neutrophils in blood of saline treated and STm challenged mice (*p* = 0.75 and *p* = 0.85, respectively; Figure 7A).

## 4. Discussion

This work extensively characterized the induction and the persistence of the immune response elicited in mice by Alhydrogel formulated *S.* Typhimurium GMMA. Moreover, upon systemic challenge with a virulent STm strain, immunized animals showed a reduction of both the bacterial load in different compartments and neutrophils in blood.

Our results show that STmGMMA/Alhydrogel induces a persistent B cell response, characterized by (i) high levels of O:4,5-specific serum IgM and IgG after the primary immunization, that are efficiently boosted and maintained for at least 28 weeks; (ii) O:4,5-specific IgG in fecal samples and intestinal washes, positively correlating with serum IgG levels; (iii) long-lasting B cell response with long-lived PCs in the spleen and bone marrow, and O:4,5-specific memory B cells in the spleen. Moreover, after i.v. challenge with a virulent African strain of STm, weight loss and bacterial load were reduced and the clinical score increased in immunized mice compared to saline treated mice, and a lower systemic recruitment of neutrophils, possibly due to the lower concentration of the granulocyte chemotactic factor G-CSF, was observed.

STm i.v. challenge in mice was employed due the capacity of STm to cause bacteremia in humans, particularly in African children aged 4–16 months [45], and the African pathotype ST313 strain D23580 is adapted to a systemic lifestyle [48]. In addition, i.v. challenge is an established animal model of infection causing a disease that resembles typhoid fever in humans [49,50]. Systemic infection suggests that continuous bacterial spread to new infection foci and host phagocytes is an essential trait in the virulence of *Salmonella* during systemic infections [51]. The control of infection though antibody mediated clearing of the bacteria has been shown in African children [52,53].

Due to their intrinsic features, GMMA are self-adjuvanting OAg delivery systems, presenting a plethora of PAMPs to the immune systems able to activate different arms of innate and adaptive responses and various effector mechanisms. Moreover, GMMA-based vaccines can be differentiated from traditional glycoconjugate and live attenuated vaccines for the simplicity and cost-effectiveness of the manufacturing process. This makes the GMMA technology an innovative platform approach well suited for the development of vaccines against neglected infectious disease for low- and middle- income countries, where high cost of production can be a critical limit for the vaccine implementation [40,54,55]. The O-antigen is considered the primary immunogenic component in GMMA. From the serotyping scheme and structure, it is expected that a STmGMMA vaccine induces antibodies specific for group B O-antigen and will react with other group B *S. enterica* serovars (e.g., Derby, Saintpaul, Heidelberg, and Paratyphi B), although they do not have H antigen commonality. Additionally, the OAg on the surface of bacteria or GMMA provides a shield which protects the bacteria from outer membrane protein specific antigens (unpublished data). A similar study has been done looking at the role of OAg and protein in *S. sonnei* GMMA [56].

The induction of persistent antibody responses against the main immunodominant serovar specific OAg [21,23,24,34,57,58] and porins [14,59,60,61,62] are recognized as critical attributes of a candidate vaccine for the control of STm infections. This is the first study that clearly characterizes the B cells response following systemic immunization with STmGMMA/Alhydrogel candidate vaccine, showing the long-term persistence of serum antibodies, the presence of local immune response in the intestine, and the generation of memory B cells and long-lived PCs. In this study we showed that STmGMMA/Alhydrogel elicited long-lasting antigen-specific IgM and IgG response with significantly higher levels compared to control mice, that persisted up at least to 10 weeks from primary immunization and 28 weeks after boosting; differently from the known short lasting, T independent antibody kinetics of plain polysaccharide vaccines [33]. The functional activity of antibodies generated against *S.* Typhimurium ST313 strain D23580 was observed in STmGMMA immunized mice compared to control group. IgM is known to be the most bactericidal of all antibody classes both in mice and humans [63,64,65,66] and in response to STmGMMA/Alhydrogel immunization, IgM levels increased after boosting with a kinetics similar to IgG. At the same time, we demonstrated the presence of memory IgM+ and IgG+ antigen specific cells, that upon in vitro reactivation, differentiated into antigen-specific ASCs. A hypothesis for the high presence of IgM producing cell is the ability of STmGMMA/Alhydrogel to induce a strong B1b cell response that in turn is associated mainly with the induction of IgM response. The critical involvement of B1b cells in antibody response against surface protein antigens of *Salmonella* was previously observed in wild type mice and confirmed in T-cell deficient mouse strains [60]. Different antigens derived from pathogens, such as pneumococcal polysaccharide and the Vi capsular polysaccharide from *S.* Typhi, are known to induce B1b responses [61,67] and, due to their ability to target protective immunity, are currently used as vaccines in humans.

The induction following systemic immunization of a local antibody response at the intestinal level, which persists over time, is of particular importance considering that this is the site of *Salmonella* entry and the main target of infection [68]. In fecal samples, we observed that IgG levels were maintained for at least 10 weeks from primary immunization and 28 weeks following boosting. Interestingly, a positive correlation of the IgG concentration in intestinal washes and fecal samples was observed underlining the critical importance of fecal samples as a tool to monitor the intestinal immune response, avoiding the sacrifice of animals. The presence of specific IgG in fecal samples reflected increased IgG in sera. These findings, suggesting passive diffusion of IgG from circulating blood to intestinal compartment or the dissemination of circulating PCs in lamina propria of the intestine, highlight the ability of parenteral immunization with STmGMMA/Alhydrogel to induce an antibody response in local compartment.

Besides the deep characterization of the immune response elicited by STmGMMA/Alhydrogel, we studied the impact of immunization on systemic STm infection, and the modulation of the early immune response in challenged mice. Challenge studies demonstrated a significant reduction of the bacterial load in blood, spleen and liver samples of mice previously immunized with STmGMMA/Alhydrogel compared to saline treated and infected mice, and this was observed both in mice challenged after 2 or 14 weeks following the booster immunization. Parenteral immunization was also able to reduce weight loss of animals after challenge, a critical clinical parameter of disease in the mouse model of infection [69,70]. Of note, the previously published data [19] used STmGMMA containing only the deletion of *tolR*, whereas we utilized the STmGMMA which also contained the modification of the lipid A (eg, Δ*msbB* and Δ*pagP*) and represent the GMMA to be evaluated in human clinical trials.

Strong involvement of B cells in the protection against STm has been previously shown using B-cell-deficient mice [59,60] or adoptive transfer studies [71,72]. The opsonic activity of antibodies response is also documented to be required for phagocytosis, oxidative burst function, and cellular killing of *Salmonella* by murine macrophages [73]. The protective role for both antibodies and the involvement of complement mediated bactericidal activity following infection was also demonstrated in humans with culture confirmed iNTS disease [14,45,52,58].

The main mechanism that warrants the maintenance of high levels of protective serum antibodies and the consequent protection from infection involves B cell response through the long-term survival of antibody-producing plasma cells in bone marrow niches. In the present study, we characterized the nature of the B-cell response to STmGMMA/Alhydrogel in terms of B-cell subpopulations and effector function. Long-lived plasma cells are antibody-secreting cells, developed within the germinal center reaction, that migrate into survival niches in the BM where they can survive for a long time [74]. We demonstrated that STmGMMA/Alhydrogel immunization induced the presence of long-lived PCs in spleen and in bone marrow, which were detected 28 weeks after booster immunization. Moreover, we have also demonstrated the persistence in the spleen of memory antigen-specific B cells, that were rapidly reactivated in vitro, differentiating into IgG^+^ and IgM^+^ ASC.

Taken together, these data demonstrate the ability of STmGMMA/Alhydrogel to elicit a long-term persistent humoral and cellular, local and systemic, immune responses against *S.* Typhimurium, and the reduction of bacterial load in different in vivo compartments following systemic challenge with a virulent strain of STm. This study further supports the clinical development of an iNTS candidate vaccine containing STmGMMA/Alhydrogel.

## Data Availability

Data available upon request.
